# PRMT5 promotes colorectal cancer growth by interaction with MCM7

**DOI:** 10.1111/jcmm.16436

**Published:** 2021-03-06

**Authors:** Xiangwei Li, Xin Wang, Jiahui Zhao, Jian Wang, Jingjing Wu

**Affiliations:** ^1^ Department of Pathology & Pathophysiology, and Department of Colorectal Surgery of the Second Affiliated Hospital Zhejiang University School of Medicine Hangzhou China; ^2^ Department of Pathology Sir Run Run Shaw Hospital Zhejiang University School of Medicine Hangzhou China; ^3^ Department of Colorectal Surgery and Oncology the Second Affiliated Hospital Zhejiang University School of Medicine Hangzhou China

**Keywords:** colorectal cancer, MCM7, PRMT5

## Abstract

Protein arginine methyltransferase 5 (PRMT5) is a type of methyltransferase enzyme that can catalyse arginine methylation of histones and non‐histone proteins. Accumulating evidence indicates that PRMT5 promotes cancer development and progression. However, its function in colorectal cancer (CRC) is poorly understood. In this study, we revealed the oncogenic roles of PRMT5 in CRC. We found that PRMT5 promoted CRC cell proliferation, migration and invasion in vitro and in vivo. We identified minichromosome maintenance‐7 (MCM7) as the direct PRMT5‐binding partner. A co‐immunoprecipitation (co‐IP) assay indicated that PRMT5 physically interacted with MCM7 and that the direct binding domain was located between residues 1‐248 in MCM7. In addition, our results from analysis of 99 CRC tissues and 77 adjacent non‐cancerous tissues indicated that the PRMT5 and MCM7 expression levels were significantly higher in CRC tissues than in control tissues, which was further confirmed by bioinformatic analysis using TCGA and GEO datasets. We also found that MCM7 promoted CRC cell proliferation, migration and invasion in vitro. Furthermore, we observed that increased PRMT5 expression predicted unfavourable patient survival in CRC patients and in the subgroup of patients with a tumour size of ≤5 cm. These data suggested that PRMT5 and MCM7 might be novel potential targets for the treatment of CRC.

## 1 INTRODUCTION

Colorectal cancer (CRC) is one of the most prevalent malignancies and a leading cause of cancer‐related death worldwide.^1^ Distant metastasis accounts for decreased patient survival rates. Due to early diagnosis and treatment of CRC, patient survival rates have been significantly improved, while distant metastasis remains the major cause of death in patients with CRC.^2^ Numerous studies have reported that CRC is closely related to genetic alterations, chronic inflammation, the intestinal microbiota, diet and lifestyle.^3^, ^4^ Specifically, the accumulation of genetic mutations, including APC, β‐catenin, TP53 and KRAS mutations, contributes to CRC development and progression.^2^, ^3^ Although increasing progress is being made in understanding the molecular mechanisms of CRC, the exact causes are still not well understood. Therefore, there is a pressing need to understand the molecular pathogenesis of CRC.

Protein arginine methyltransferase 5 (PRMT5), a member of the PRMT family of enzymes, is able to catalyse the monomethylation and symmetric dimethylation of arginine residues in several cytoplasmic and nuclear substrates.^5^ PRMT5 not only methylates histone H2A, H3 and H4 but also targets non‐histone proteins such as EGFR and spliceosomal Sm proteins.^6^, ^7^ It is highly conserved and emerging as an important epigenetic factor in signal transduction, thereby affecting chromatin remodelling, cell differentiation, senescence, cell cycle, proliferation, apoptosis and transformation in cancer.^8^, ^9^ Moreover, upregulation of PRMT5 has been reported in various human malignancies, including gastric cancer, breast cancer, lung cancer and ovarian cancer.^8^, ^9^ However, currently, the molecular mechanisms by which PRMT5 promotes CRC development and the role of PRMT5 in CRC remain elusive. This is an area deserving further investigation.

In the current study, we identified PRMT5 as a potential target in CRC development and progression. We explored the oncogenic roles of PRMT5, and our results showed that PRMT5 induced CRC cell proliferation, migration and invasion in vitro and in vivo. We revealed the physical interaction between PRMT5 and the minichromosome maintenance‐7 (MCM7) protein with a co‐immunoprecipitation (co‐IP) assay, and the direct binding domain was found to be located between residues 1‐248 in MCM7. The proliferative, migratory and invasive properties of CRC cells were greatly impaired after MCM7 silencing in vitro. Furthermore, we evaluated the PRMT5 and MCM7 protein levels in 99 CRC and 77 adjacent non‐cancerous tissues by immunohistochemical (IHC) analysis. These results indicated that PRMT5 and MCM7 were upregulated in CRC compared with normal control tissues. Our results also showed a positive correlation between PRMT5 and MCM7 expression. In addition, we found that PRMT5 could be used to predict for CRC patient outcomes and that patients with high PRMT5 expression among total CRC patients and in the subgroup with a tumour size of ≤5 cm had unfavourable outcomes. In addition, bioinformatic analysis of CRC data from TCGA and GEO verified the oncogenic characteristics of PRMT5 and MCM7 in CRC. In summary, our data provided new insight into PRMT5 and MCM7 as potential diagnostic and therapeutic targets in CRC.

## 2 MATERIALS AND METHODS

### 2.1 Patients and tissue specimens

A total of 99 CRC and 77 adjacent normal samples were collected from the National Engineering Center for Biochip at Shanghai (SBC). All CRC tissues were diagnosed histologically. This study was permitted and approved by the Ethics Committee at Zhejiang University.

### 2.2 Cell lines and cell culture

The human CRC cell lines RKO, HT29, HCT8 and HCT116 and the human embryonic kidney cell line HEK293T were obtained from the American Type Culture Collection (ATCC, Manassas, VA, USA). HT29, HCT8 and HCT116 cells were maintained in RPMI‐1640 medium (Gibco, Grand Island, NY, USA) supplemented with 10% foetal bovine serum (Gibco), while RKO and HEK293T cells were grown in Dulbecco's modified Eagle's medium (Gibco) containing 10% foetal bovine serum.

### 2.3 Generation of stable PRMT5 overexpression or knockdown cells by lentiviral infection

The cDNA encoding the full‐length PRMT5 open reading frame (ORF) was amplified and engineered into the lentiviral vector pHBLV with a luciferin reporter; the sequences of the primers used for the generation of constructs are listed in Table S1. A short hairpin RNA (shRNA) construct for human PRMT5 knockdown was generated using the pLKO.1 lentiviral vector. Empty vector was used as the negative control. Subsequently, cells were infected with lentivirus‐containing supernatant for 24 hours and were then selected for 2 weeks in the presence of 4 μg/mL puromycin (Sigma, St. Louis, MO, USA).

### 2.4 Transient transfections with plasmids and oligonucleotides

The full‐length ORF or sequences of various truncation mutants of PRMT5 or MCM7 were amplified and inserted into the pcDNA3.1‐Myc or pcDNA3.1‐FLAG vector. The sequences of the primers used for the generation of constructs are listed in Table S1. Empty vector was used as the negative control. Two small interfering RNAs (siRNAs) against PRMT5 (5′‐ACCGCUAUUGCACCUUGGA‐3′ and 5′‐GCCCAGUUUGAGAUGCCUU‐3′) and MCM7 (5′‐GAUGUCCUGGACGUUUACA‐3′ and 5′‐GAGUUGGUGGACUCAAUUU‐3′) were designed and provided by GenePharma (Shanghai, China), and a scrambled siRNA sequence was used as the negative control. Transient transfections of plasmids and oligonucleotides were carried out using Lipofectamine 3000 (Invitrogen, Carlsbad, CA, USA) according to the manufacturer′s instructions.

### 2.5 RNA isolation and qRT‐PCR analysis

Total RNA was isolated using Trizol (Invitrogen) and subsequently reverse transcribed into cDNA using PrimeScript RT reagent kit (TaKaRa, Tokyo, Japan). Then qPCR was performed with SYBR green (Roche, Basel, Switzerland) and normalized to GAPDH according to manufacturer protocols. The sequences of the primers are listed in Table S2.

### 2.6 Animal studies and tail vein transplantation

Control or PRMT5‐overexpressing HT29 cells (5 × 10^5^ cells in 100 μL of PBS) were injected into the tail veins of 5‐ to 6‐week‐old BALB/c nude mice. Six weeks after injection, mice were anaesthetized with isoflurane (Sigma) and intraperitoneally injected with D‐Luciferin substrate (PerkinElmer, Waltham, MA, USA) prior to analysis of bioluminescence signals using an in vivo imaging system (IVIS) Spectrum System (Caliper Life Sciences, Waltham, MA, USA). After mice were sacrificed, lungs and brains were surgically excised and processed for staining with haematoxylin and eosin (H&E). All mouse studies and procedures were conducted in accordance with the guidelines established by the Animal Care and Use Committee of Zhejiang University.

### 2.7 Co‐IP assay

After the indicated cells were lysed in lysis buffer on ice for 30 minutes, the cleared cell lysates were incubated with beads conjugated to the indicated antibody or IgG control (Dakewe, Shenzhen, China) overnight at 4°C with gentle agitation. The precipitated samples were washed three times and further analysed by western blotting using the indicated antibodies.

### 2.8 Statistical analysis

All results are presented as the mean ± standard deviation (SD) or mean ± standard error of the mean (SEM) values. Student's *t* test was used for comparisons between two groups, and one‐way ANOVA was used for comparisons among three or more groups. All statistical analyses were performed using SPSS 17.0 software (Chicago, IL, USA) or GraphPad Prism 7.0 software (San Diego, CA, USA). *P* < .05 was considered statistically significant.

## 3 RESULTS

### 3.1 PRMT5 promotes the proliferation, migration and invasion of CRC cells in vitro and in vivo

**FIGURE 1:**
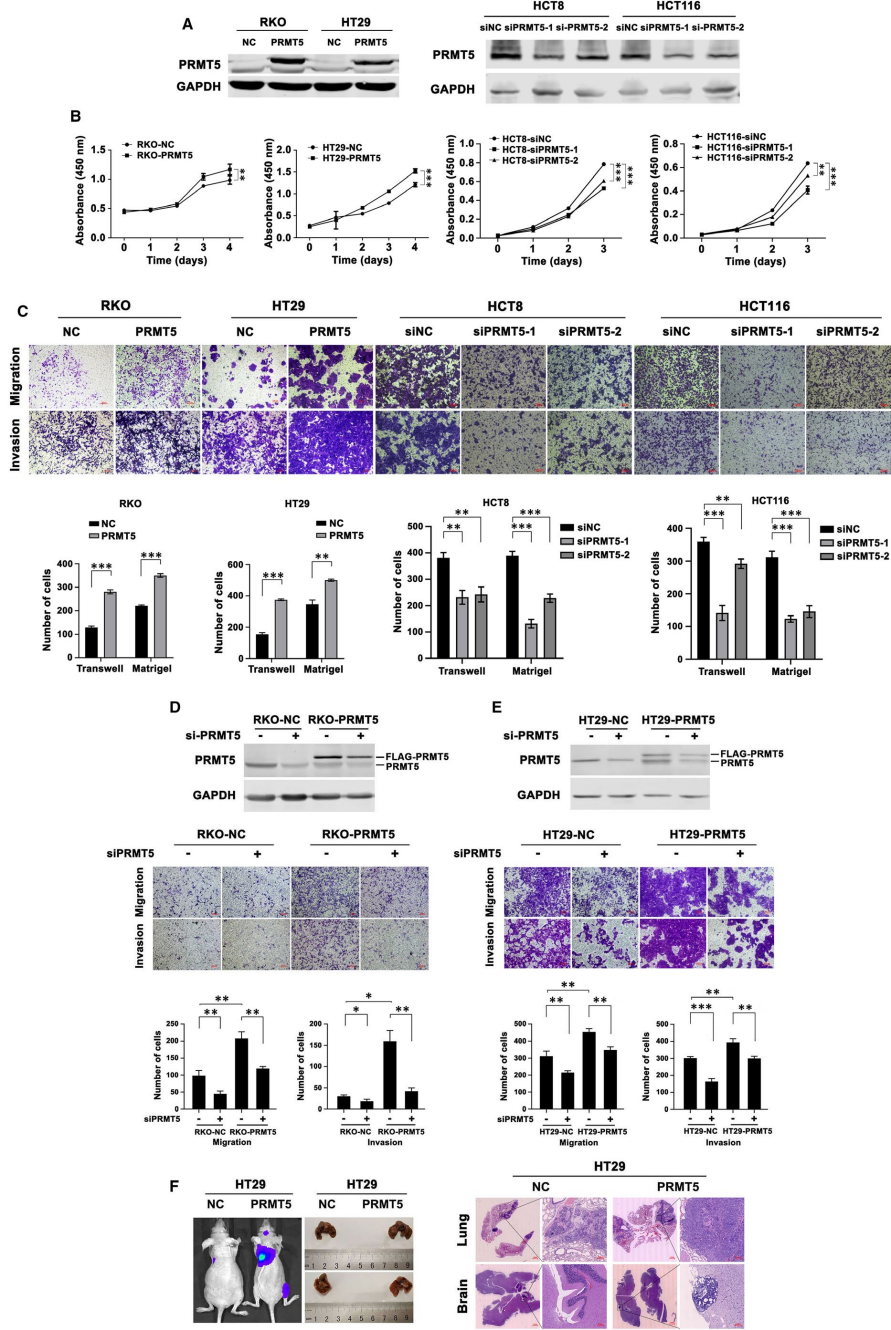
PRMT5 promotes the proliferation, migration and invasion of CRC cells in vitro and in vivo. A, Western blot analysis of PRMT5 levels in RKO and HT29 cells stably transfected with the control or PRMT5 expression vector (left panel; RKO‐NC/RKO‐PRMT5 and HT29‐NC/HT29‐PRMT5, respectively) and in HCT8 and HCT116 cells transfected with the control siRNA or either of two PRMT5‐specific siRNAs (right panel; HCT8‐NC/HCT8‐siPRMT5 and HCT116‐NC/HCT116‐siPRMT5, respectively). GAPDH was used as the loading control. B, CCK‐8 cell proliferation assay of the indicated cells at the indicated time points. All experiments were performed in triplicate, and the results are presented as the mean ± SD values. **P < .01; ***P < .001. C, Images (upper) and quantification (bottom) of migrated and invaded cells in the migration and invasion assays. Scale bar: 100 μm. All data are presented as the mean ± SD of three independent experiments. **P < .01; ***P < .001. D‐E, (D) RKO‐NC/RKO‐PRMT5 and (E) HT29‐NC/HT29‐PRMT5 cells were transfected with scramble siRNA or siPRMT5 for 48 hours prior to western blot analysis and migration and invasion assays. Upper panel: PRMT5 protein levels were verified by western blotting. GAPDH was used as the loading control. Middle panel: representative photographs of the migrated and invaded cells in the migration and invasion assays. Scale bar: 100 μm. Bottom panel: the bars show the mean ± SD of experiments performed in triplicate. *P < .05; **P < .01; ***P < .001. F, Representative bioluminescence images (left), gross images of excised lungs (middle) and images of H&E‐stained lung and brain sections (right) from each group of mice injected with HT29‐NC and HT29‐PRMT5 cells via the tail vein. Scale bars: 1 mm or 100 μm

To investigate the functions of PRMT5 in CRC, we used FLAG‐tagged PRMT5 lentiviruses to exogenously overexpress PRMT5 in RKO and HT29 cells, which have relatively low expression levels of endogenous PRMT5 compared with other CRC cell lines. We used two siRNAs targeting PRMT5 to knock down PRMT5 in HCT8 and HCT116 cells, which have strong endogenous expression of PRMT5. The protein levels of PRMT5 were confirmed by western blotting (Figure 1A). To determine whether PRMT5 contributes to CRC cell malignancy, we compared the proliferation of these cells by a CCK‐8 assay. As described in Figure 1B, PRMT5 overexpression accelerated the growth of RKO and HT29 cells, whereas PRMT5 silencing impaired the growth of HCT8 and HCT116 cells. Furthermore, the Boyden chamber assay revealed that PRMT5 overexpression drastically enhanced cell migration and invasion (Figure 1C). By contrast, cells with PRMT5 knockdown exhibited significantly reduced migration and invasion capacities compared with the corresponding controls (Figure 1C). This observation was further confirmed by alterations in the expression profiles of proliferation‐, migration‐ and invasion‐related genes. HCT8 and HCT116 cells with stable PRMT5 knockdown were established and the knockdown efficiency was examined by western blot analysis (Figure S1A). Quantitative reverse transcription‐polymerase chain reaction (qRT‐PCR) analysis showed that in RKO and HT29 cells, ectopic expression of PRMT5 greatly decreased *E‐cadherin* levels, whereas it increased *Vimentin* and *c‐Myc* expression levels (Figure S1B‐E). On the other hand, stable shRNA‐mediated knockdown of PRMT5 induced the expression of *E‐cadherin* but reduced the expressions of *Vimentin* and *c‐Myc* in HCT8 and HCT116 cells (Figure S1B‐E).

Next, RKO cells without or with PRMT5 overexpression (RKO‐NC and RKO‐PRMT5 cells, respectively) were transfected with control siRNA or siRNA targeting PRMT5 (si‐PRMT5). The efficiency of PRMT5 silencing was confirmed by western blotting (Figure 1D), and cells were then subjected to a Boyden chamber assay to compare their migratory and invasive abilities. As expected, RKO‐PRMT5 cells displayed significantly enhanced migration and invasion compared with RKO‐NC cells, whereas silencing PRMT5 caused a significant reduction in the migratory and invasive capacities (Figure 1D). Notably, similar results were also obtained in HT29 cells (Figure 1E).

To validate the above results in vivo, we implanted luciferase‐labelled HT29 cells with or without PRMT5 overexpression into nude mice via the lateral tail vein to examine the effect of PRMT5 on metastasis. Metastasis formation was monitored by bioluminescence imaging with an IVIS system. Consistent with the role of PRMT5 in vitro, we found that overexpression of PRMT5 significantly promoted distant metastasis to the lungs and brain (Figure 1F). After 6 weeks, all mice were euthanized, and lungs and brains were collected. Intriguingly, the tumours in mice implanted with PRMT5‐overexpressing cells exhibited a more aggressive phenotype with the development of more and larger metastatic foci in the lungs and brains, as shown by macroscopic inspection and histologic analysis (Figure 1F). Collectively, the results of these in vitro and in vivo studies suggested that PRMT5 promoted the proliferation, invasion and metastasis of CRC cells.

### 3.2 PRMT5 directly interacts with MCM7, and MCM7 promotes the proliferation, migration and invasion of CRC cells in vitro

**FIGURE 2:**
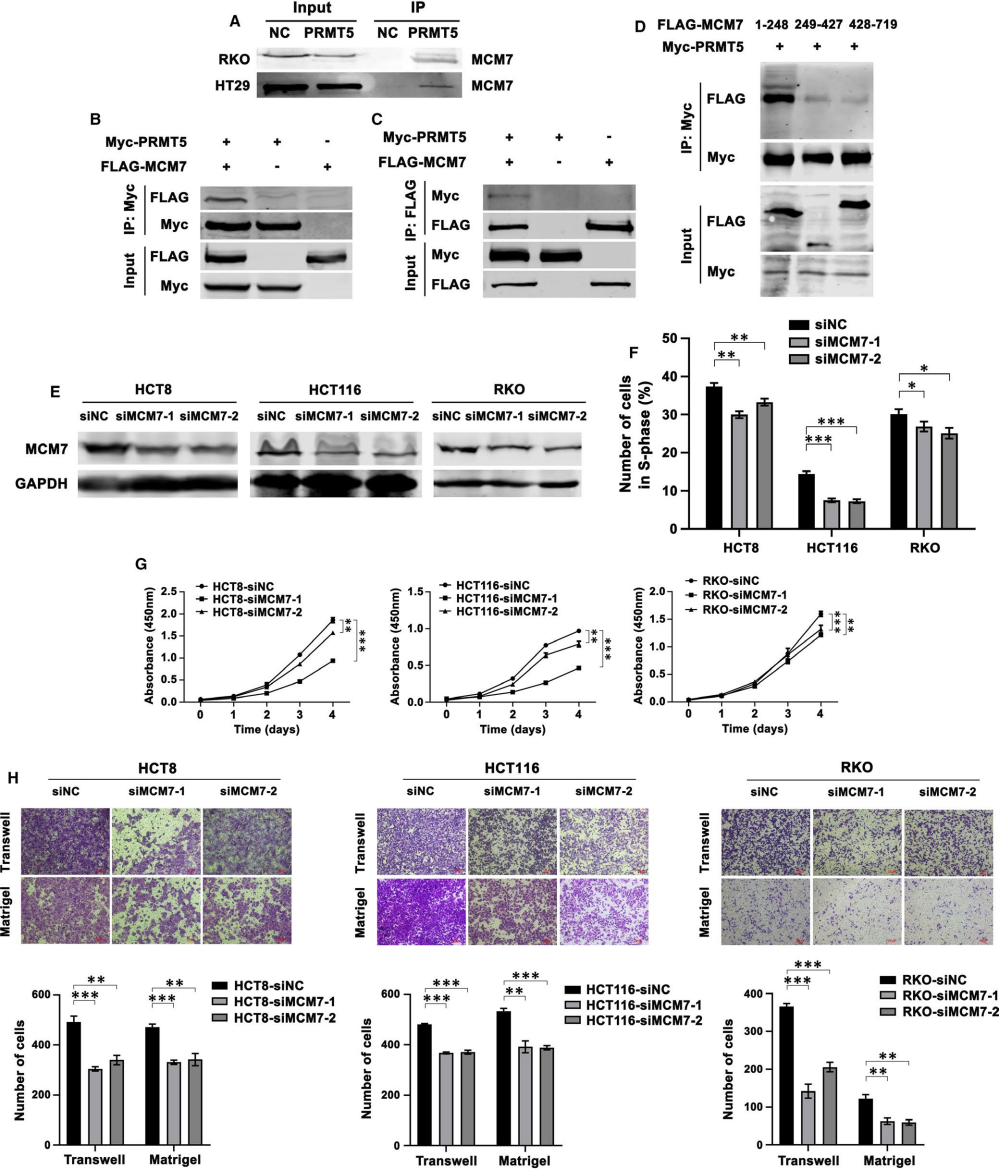
PRMT5 directly interacts with MCM7, and MCM7 promotes proliferation, migration and invasion of CRC cells in vitro. A, Co‐IP analysis of the interaction between FLAG‐PRMT5 and MCM7 in FLAG‐tagged RKO‐NC/RKO‐PRMT5 and HT29‐NC/HT29‐PRMT5 cells with an anti‐FLAG antibody. B‐C, Co‐IP analysis of the interaction between PRMT5 and MCM7 in HEK293T cells transiently transfected with full‐length Myc‐PRMT5 and/or FLAG‐MCM7 vector with an (B) anti‐Myc or (C) anti‐FLAG antibody. D, Co‐immunoprecipitation of PRMT5 and MCM7 in HEK293T cells transiently transfected with vectors expressing full‐length Myc‐PRMT5 and various FLAG‐MCM7 truncations with an anti‐Myc antibody. E, western blot analysis verifying the efficiency of MCM7 knockdown in HCT8, HCT116 and RKO cells transfected with the scramble control siRNA or either of two siRNAs targeting MCM7 for 48 h. GAPDH was used as the loading control. F, Flow cytometric analysis of the S‐phase proportions of the indicated cells transfected with the scramble control siRNA or siMCM7. The data are presented as the mean ± SD of experiments performed in triplicate. *P < .05; **P < .01; ***P < .001. G, CCK‐8 cell proliferation assay of the indicated cells transfected with the scramble control siRNA or siMCM7 at the indicated time points. All experiments were performed in triplicate, and the results are presented as the mean ± SD values. **P < .01; ***P < .001. H, Images (upper) and quantification (bottom) of the migrated and invaded cells among the indicated cells transfected with the scramble control siRNA or siMCM7, as assessed by migration and invasion assays. Scale bar: 100 μm. All data are presented as the mean ± SD of three independent experiments. **P < .01; ***P < .001

To clarify the molecular mechanism by which PRMT5 stimulates the aggressive properties of CRC, we performed co‐IP and mass spectrometry (MS) analysis to identify the potential PRMT5‐interacting proteins in HT29‐PRMT5 cells. MCM7, WDR77 and other proteins were identified as potential binding partners of PRMT5 (Table S3). We next sought to analyse the direct interaction between PRMT5 and MCM7. We first performed co‐IP on FLAG‐tagged RKO‐NC/RKO‐PRMT5 and HT29‐NC/HT29‐PRMT5 cells. Immunoprecipitates were obtained from cell lysates incubated with an anti‐FLAG antibody and were then analysed by western blotting using an anti‐MCM7 antibody. Figure 2A shows that PRMT5 physically interacted with MCM7. Then, this interaction was further confirmed by a co‐IP assay in HEK293T cells. HEK293T cells were transiently transfected with Myc‐tagged PRMT5 and/or FLAG‐tagged MCM7, and the cell lysates were then subjected to co‐IP with an anti‐Myc antibody followed by immunoblotting with an anti‐FLAG or anti‐Myc antibody. As shown in Figure 2B, our results revealed that exogenous Myc‐tagged PRMT5 was strongly co‐precipitated with exogenous FLAG‐tagged MCM7 in HEK293T cells. Similarly, the direct interaction between PRMT5 and MCM7 was inversely confirmed by immunoprecipitation with an anti‐FLAG antibody in HEK293T cells (Figure 2C). To identify the specific domain in MCM7 that binds to PRMT5, truncation mutants of FLAG‐tagged MCM7 (residues 1‐248, 249‐427 or 428‐719) were expressed together with full‐length Myc‐tagged PRMT5 in HEK293T cells. As shown in Figure 2D, a co‐IP assay using an anti‐Myc antibody revealed that the fragment spanning amino acids 1‐248, but not the fragments spanning amino acids 249‐427 and 428‐719, was required for the binding of MCM7 to PRMT5. To evaluate the roles of MCM7 in CRC, we silenced MCM7 expression using two siRNAs in HCT8, HCT116 and RKO cells. The knockdown efficiency was confirmed by western blotting (Figure 2E). Flow cytometric analysis was employed to investigate the roles of MCM7 in the process of cell cycle deregulation. As shown in Figure 2F, MCM7 knockdown decreased the proportions of S‐phase cells among HCT8, HCT116 and RKO cells. Consistent with this finding, MCM7 knockdown significantly decreased cell growth, as shown by cell proliferation assays (Figure 2G). Boyden chamber assays demonstrated that silencing MCM7 by siRNA transductions impaired the migratory and invasive abilities of HCT8, HCT116 and RKO cells (Figure 2H). To confirm the functions of MCM7 identified above, qRT‐PCR analysis was performed to determine the mRNA levels of proliferation‐, migration‐ and invasion‐related genes after MCM7 knockdown in a panel of HCT8, HCT116 and RKO cells. Expression of *E‐cadherin* was significantly induced upon loss of MCM7, whereas the expression levels of *Vimentin* and *c‐Myc* were reduced (Figure S2A‐D). Taken together, these results indicated that PRMT5 directly interacted with MCM7, which promoted cell proliferation, migration and invasion in vitro. In addition, the distinct binding domain in MCM7 was identified as the regions between residues 1‐248.

### 3.3 PRMT5 and MCM7 expression levels are elevated in clinical samples, and high expression of PRMT5 is associated with poor prognosis in CRC

**FIGURE 3:**
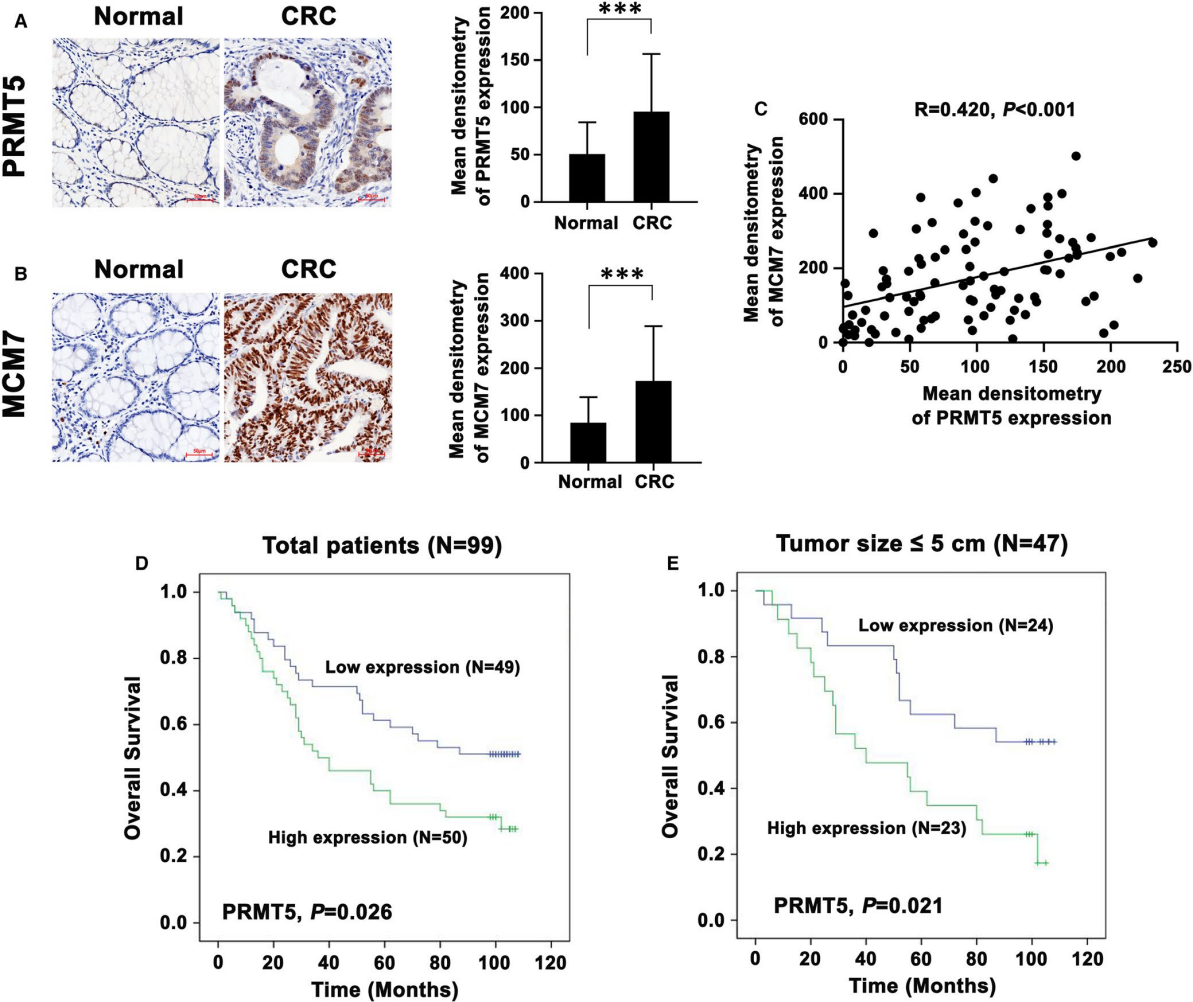
PRMT5 and MCM7 expression levels are elevated in clinical samples, and high expression of PRMT5 is associated with poor prognosis in CRC. A‐B, Representative images (left) and quantification (right) of IHC staining for (A) PRMT5 and (B) MCM7 proteins in 99 CRC tissues and 77 adjacent non‐cancerous tissues. Scale bar: 50 μm. The data are presented as the mean ± SD values. ***P < .001. C, Dot plot showing the correlation between PRMT5 and MCM7 protein expression in all CRC tissues (*R* = .420, *P* < .001). D, Kaplan‐Meier analysis of overall survival comparing CRC patients (N = 99) stratified according to the PRMT5 expression level (high PRMT5 expression vs. low PRMT5 expression, *P* = .026). E, Kaplan‐Meier analysis of overall survival comparing CRC patients with a tumour size of ≤5 cm (N = 47) stratified according to the PRMT5 expression level (high PRMT5 expression vs. low PRMT5 expression, *P* = .021)

To detect PRMT5 expression in human CRC samples and determine its potential association with clinical outcomes, we carried out IHC assays and used Image‐Pro Plus 6.0 software to analyse PRMT5 expression in 99 CRC and 77 adjacent non‐cancerous tissues. Importantly, PRMT5 protein levels were found to be significantly higher in CRC samples than in normal control tissues (Figure 3A). Consistent with this pattern, MCM7 expression was elevated in tumour tissues compared with normal controls (Figure 3B). Importantly, as shown in Figure 3C, the protein level of PRMT5 was significantly associated with that of MCM7 in CRC samples (*R* = .420, *P* < .001), further suggesting the correlation and interaction between PRMT5 and MCM7 in CRC.

To further evaluate the prognostic value of PRMT5 in CRC, the patients were divided into the low and high expression groups, and overall survival analysis was conducted. The Kaplan‐Meier curves showed that compared with patients with low PRMT5 levels, patients with high PRMT5 levels were characterized by poor overall survival in CRC (*P* = .026, Figure 3D). Then, we performed a subgroup analysis. After stratification of all patients by tumour size (>5 cm or ≤5 cm), patients with low PRMT5 expression had a survival advantage in the subgroup with a tumour size of ≤5 cm (*P* = .021, Figure 3E). Collectively, these data demonstrated that PRMT5 and MCM7 were upregulated in CRC and that a high protein level of PRMT5 was strongly associated with a poor clinical outcome in CRC.

### 3.4 *PRMT5* and *MCM7* mRNA expression is upregulated in CRC samples in TCGA and GEO datasets

**FIGURE 4:**
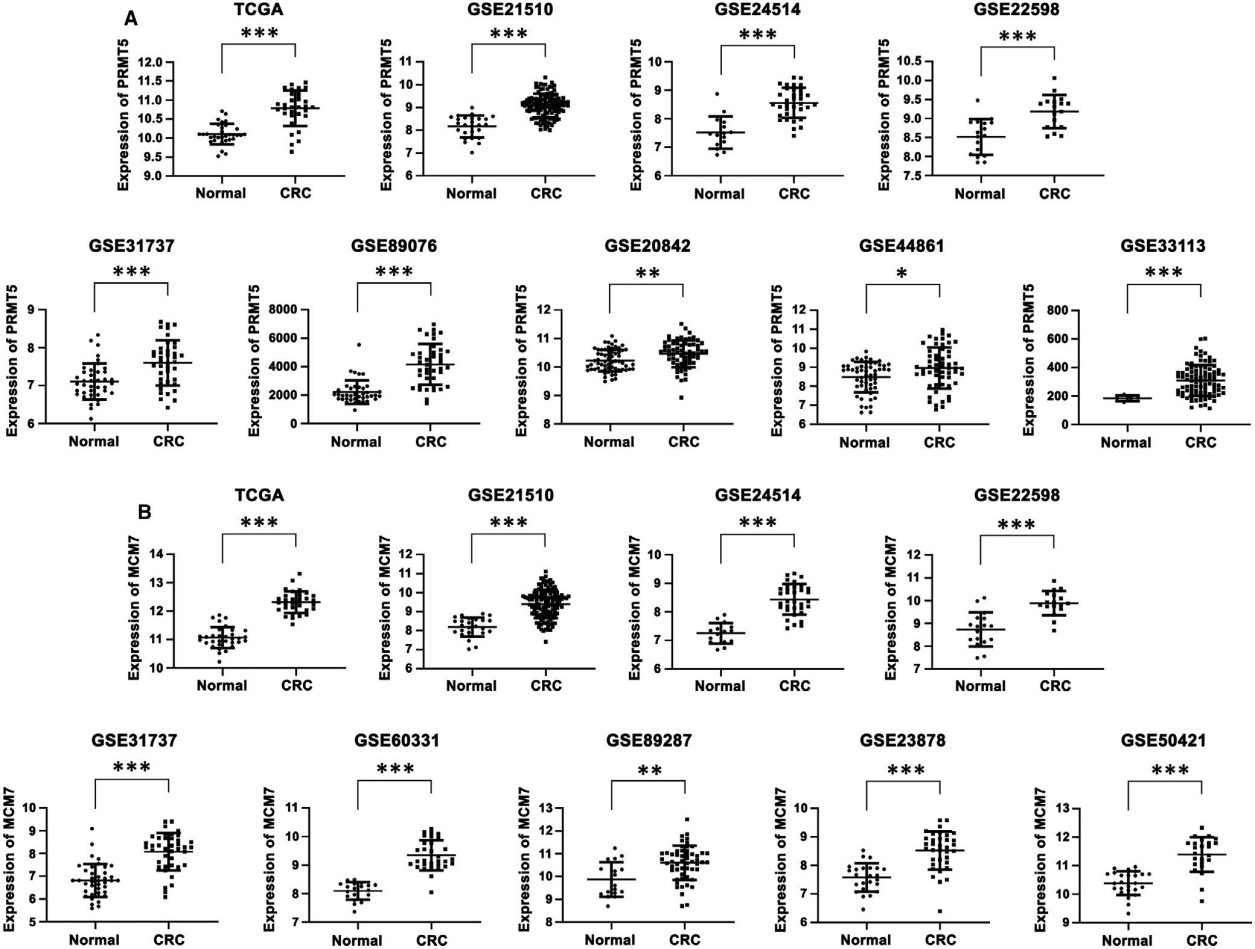
*PRMT5* and *MCM7* mRNA expression is upregulated in CRC samples in TCGA and GEO datasets. A, Bioinformatic analysis of *PRMT5* mRNA expression in CRC and non‐cancerous tissues in the TCGA, GSE21510, GSE24514, GSE22598, GSE31737, GSE89076, GSE20842, GSE44861 and GSE33113 datasets. B, Bioinformatic analysis of *MCM7* mRNA expression in CRC and non‐cancerous tissues in the TCGA, GSE21510, GSE24514, GSE22598, GSE31737, GSE60331, GSE89287, GSE23878 and GSE50421 datasets. The data are presented as the mean ± SD values. *P < .05; **P < .01; ***P < .001

To validate the previous observations, we employed public data sets from TCGA and GEO to evaluate *PRMT5* and *MCM7* mRNA expression in CRC and normal tissue samples. The human CRC datasets from TCGA contained 32 paired tissues.^10^ Twelve GEO datasets were used here (Table S4): GSE21510 (25 adjacent control and 123 CRC tissues),^11^ GSE24514 (15 adjacent control and 34 CRC tissues),^12^ GSE22598 (17 paired tissues),^13^ GSE31737 (40 paired tissues),^14^ GSE89076 (39 adjacent control and 41 CRC tissues),^15^ GSE20842 (65 paired tissues),^16^ GSE44861 (55 adjacent control and 56 CRC tissues),^17^ GSE33113 (6 adjacent control and 90 CRC tissues),^18^ GSE60331 (19 adjacent control and 31 CRC tissues),^19^ GSE89287 (17 adjacent control and 54 CRC tissues),^20^ GSE23878 (24 adjacent control and 35 CRC tissues)^21^ and GSE50421 (25 adjacent control and 24 CRC tissues).^22^ As shown in Figure 4A, we observed that *PRMT5* mRNA levels were significantly increased in CRC compared with non‐cancerous tissues in the TCGA (*P* < .001), GSE21510 (*P* < .001), GSE24514 (*P* < .001), GSE22598 (*P* < .001), GSE31737 (*P* < .001), GSE89076 (*P* < .001), GSE20842 (*P* < .01), GSE44861 (*P* < .05), and GSE33113 (*P* < .001) datasets. Additionally, we analysed *MCM7* mRNA expression in the TCGA and GEO data sets. As shown in Figure 4B, *MCM7* mRNA levels were remarkably increased in CRC compared with non‐cancerous tissues in the TCGA (*P* < .001), GSE21510 (*P* < .001), GSE24514 (*P* < .001), GSE22598 (*P* < .001), GSE31737 (*P* < .001), GSE60331 (*P* < .001), GSE89287 (*P* < .01), GSE23878 (*P* < .001) and GSE50421 (*P* < .001) data sets. Collectively, our data demonstrated that *PRMT5* and *MCM7* mRNA levels were high in CRC tissues in public data sets and might be involved in the pathogenesis of CRC.

## 4 DISCUSSION

PRMT family proteins are a large class of enzymes that are well characterized as protein arginine methyltransferases. Based on patterns of arginine methylation, the nine mammalian PRMTs are classified into two major groups: type I and type II.^9^ PRMT5, a type II arginine methyltransferase, is frequently upregulated in diverse cancers and linked to poor prognosis in several cancers.^8^, ^9^ In addition, it influences a wide spectrum of cellular and biological processes, ranging from tumour initiation and progression to stem cell maintenance and embryo development.^8^, ^9^ There is accumulating evidence that PRMT5 plays a crucial role in CRC carcinogenesis. A study from Wei and colleagues revealed that PRMT5 interacted with p65 and substantially increased NF‐κB activity by dimethylating arginine 30 (R30) in its p65 subunit in mouse embryo fibroblasts (MEFs).^23^ Interestingly, this group found that interleukin‐1 (IL‐1) stimulation facilitated phosphorylation of PRMT5 on serine 15 (S15), which is critical for NF‐κB activation by PRMT5, thus enhancing the proliferative, migratory and anchorage‐independent growth capabilities of CRC cells.^24^ Furthermore, they developed and adapted AlphaLISA technology to identify small molecule inhibitors of PRMT5.^25^ Their work showed that PR5‐LL‐CM01 had strong potential to block PRMT5 in CRC.^26^ In addition, it has been shown that PRMT5 promotes H3R8 and H4R3 methylation in the FGFR3 and eIF4E promoters via direct binding of PRMT5 to these promoters, resulting in upregulated expression of FGFR3 and eIF4E.^27^ Rastetter et al^28^ found that PRMT5 directly interacted with and methylated CRN5, which increased TRAIL receptor sensitivity. Cho et al^29^ showed that PRMT5 methylated E2F‐1 and that depleting PRMT5 enhanced the expression of E2F‐1 by increasing its protein stability, thereby resulting in a decrease in cell growth and an increase in apoptosis. Notably, their study also revealed a negative correlation between PRMT5 and E2F‐1 expression in CRC tissues.^29^ In addition, Demetriadou et al^30^ reported that PRMT5 can be transcriptionally regulated by N‐alpha‐acetyltransferase 40 (NAA40) and found a significant positive correlation between PRMT5 and NAA40 mRNA levels in CRC. In an IHC analysis of PRMT5 in endoscopically resected early colorectal carcinoma, Pak et al^31^ found that high nuclear expression of PRMT5 can be used as a predictive marker for submucosal invasion of early CRC.

The MCM family of proteins, which is highly conserved, was originally identified for its function in minichromosome maintenance in yeast. As components of the pre‐replicative complex (Pre‐RC), six subunits (MCM2‐7) interact to form the heterohexameric MCM complex during early G1 phase.^32^ This complex is thought to exert DNA helicase activity at replication forks to subsequently facilitate the unwinding of DNA strands and the recruitment of related proteins during the initiation of DNA synthesis.^32^ Therefore, these proteins are markers of cell proliferation. DNA replication is tightly and strictly regulated to maintain genome stability through highly ordered and precise steps, while one of the hallmarks of cancer is the acquisition of replicative immortality. Therefore, dysregulation of MCM proteins is significantly associated with tumorigenesis.^32^, ^33^

As mentioned above, MCM7, known as a member of the MCM family, is a pivotal component of the MCM complex, in which, it allows the initiation of eukaryotic genome replication during G1 phase and elongation of DNA strands during S‐phase. Upregulated MCM7 expression is believed to be related to increased cell proliferation and tumour progression. A variety of MCM7‐binding partners have been identified to date. For instance, Sterner et al^34^ reported that Rb was the binding protein of MCM7 and that its interaction mediated cell growth arrest by abrogating DNA replication. However, Gladden et al^35^ revealed an association between MCM7 and the cyclin D1/CDK4 complex, which subsequently dissociated the Rb‐MCM7 complex and promoted the assembly of the pre‐RC to facilitate DNA replication. In addition, Cortez et al^36^ revealed an association between MCM7 and ATR‐interacting protein (ATRIP)‐ATR, which is involved in DNA replication damage. Emerging evidence implies an oncogenic role of MCM7 in CRC progression. In their study based on colorectal cancer patients enrolled in the Gene Expression Omnibus (GEO) database, Shi et al^37^ found that MCM7 levels were significantly elevated and gradually increased with advancing stage (stage I to IV) in CRC. Thus, MCM7 might be a CRC stage‐related gene. Intriguingly, the study by Kabir and colleagues identified *Melicope ptelefolia* (MP) hexane extract as an agent with anticancer therapeutic potential via its modulation of the expression of cancer‐associated genes. Importantly, it significantly attenuated MCM7 expression in CRC.^38^ In agreement with the roles of MCM7 in CRC, Ishibashi et al^39^ reported that positive MCM7 expression was correlated with shorter overall survival and recurrence‐free survival times in patients with Dukes C CRC. Consistent with this finding, univariate and multivariable Cox proportional hazards analyses revealed that MCM expression was an independent prognostic factor for worse RFS in Dukes C CRC patients. Moreover, a study by Pillaire and colleagues provided further support that MCM7 affects the prognosis of patients with CRC. They found that high expression of MCM7 predicted poor patient outcomes, indicating that MCM7 could be used as a prognostic biomarker in CRC.^40^ In addition, it has been shown that CRC patients with positive MCM7 but negative Ki67 expression tend to have an increased incidence of lymph node metastasis, positive distant metastasis and more advanced UICC stages, suggesting crucial roles of MCM7 in regulating CRC progression.^41^ In the present study, our data indicated that PRMT5 directly and physically interacted with MCM7 in CRC. We speculate that PRMT5 regulates the expression of MCM7. These proteins cooperate and function as an integral complex to promote CRC progression by directly or indirectly influencing the expression of target genes.

PRMT5 is known to form a complex with MEP50 and catalyse the methylation of arginine residues in histones and non‐histone proteins. PRMT5 methylation of histone H4R3 and H3R8 is generally correlated with transcriptional repression, whereas PRMT5‐catalyzed H3R2 methylation is generally correlated with transcriptional activation.^42^ Tae et al^43^ reported that BRD7 forms a complex with MEP50 and PRMT5, which elicited histone H3R8 and H4R3 methylation, resulting in transcriptional repression of the ST7 promoter. PRMT5‐mediated H3R2me2s was found to recruit WDR5 and favour H3K4 methylation, leading to transcriptional activation of target genes.^44^ PRMT5 interacted with and elicited p53 methylation in its oligomerization region, which resulted in alterations in the p53 response.^45^ In addition, PRMT5‐catalyzed methylation of KLF4 blocked its ubiquitination by pVHL, which then enhanced its stability.^46^ It is likely that PRMT5 targets SREBP1a for methylation, antagonizing its GSK‐3β‐mediated phosphorylation and FBXW7‐mediated ubiquitination, which in turn, stabilizes it.^47^ In the present study, we found that PRMT5 directly interacted with MCM7 in CRC. We speculate that there are several possible functions of the PRMT5/MCM7 interaction. First, PRMT5 is known to exert its function when complexed with MEP50/WDR77. As shown in Table S3, co‐IP and MS analysis identified WDR77 (also named MEP50) as a PRMT5‐binding protein in CRC. It is speculated that MCM7 is a direct substrate for the PRMT5/MEP50 enzyme complex. We hypothesize that PRMT5 catalyses the methylation of MCM7, an event that might alter the protein dynamics of MCM7 and then stabilize it. This hypothesis may explain why PRMT5 and MCM7 are upregulated in CRC and were found to be positively correlated in our study (Figure 3). Second, we hypothesize that MCM7 may function as a bridge between the PRMT5/MEP50 complex and its substrates. We speculate that it may facilitate PRMT5‐mediated methylation of target proteins, which in turn, activates or represses the transcription of target genes (eg E‐cadherin, Vimentin and c‐Myc) through histone modification (Figures [Link], [Link] and [Link], [Link]). Future studies should focus on clarifying these hypotheses and uncovering new mechanisms of the PRMT5/MCM7 complex in CRC.

In this study, we investigated the oncogenic roles of PRMT5 and MCM7 in CRC. We revealed a new form of molecular interplay between PRMT5 and MCM7 in the pathogenesis of CRC. A direct association between the oncoprotein PRMT5 and MCM7 was identified. We found that PRMT5 promoted cell proliferation, migration and invasion in CRC in vitro and in vivo. To elucidate the molecular mechanisms, co‐IP and MS analysis were performed, and we identified a variety of potential PRMT5‐binding proteins, including WDR77 and MCM7. Our co‐IP results confirmed that PRMT5 directly interacted with the MCM7 protein. The fragment of MCM7 spanning amino acids 1‐248 was found to be required for its interaction with PRMT5. In addition, we evaluated the expression of PRMT5 and MCM7 in a large cohort of 99 CRC and 77 adjacent non‐cancerous tissues. IHC staining demonstrated that upregulation of PRMT5 and MCM7 frequently occurred in CRC tissues compared to controls. Our results also revealed a positive correlation between the PRMT5 and MCM7 protein levels. Bioinformatic analysis using TCGA and GEO data sets confirmed the elevated levels of *PRMT5* and *MCM7* mRNA in CRC. Additionally, we found that PRMT5 could be a prognostic predictor in CRC patients. Kaplan‐Meier survival analysis indicated that individuals with high PRMT5 expression showed poor clinical outcomes among total CRC patients and in the subgroup with a tumour size of ≤5 cm. Taken together, the results of this study demonstrated that PRMT5 promotes the malignant behaviour of colorectal cancer by interacting with MCM7. In conclusion, our study supports the targeting of PRMT5 and MCM7 as novel clinical intervention strategies in CRC.

## CONFLICT OF INTEREST

All the authors declare no conflict of interest.

## AUTHOR CONTRIBUTIONS

**Xiangwei Li:** Data curation (lead); Formal analysis (lead); Methodology (lead); Writing‐review & editing (equal). **Xin Wang:** Data curation (equal); Methodology (equal); Writing‐review & editing (equal). **Jiahui Zhao:** Data curation (equal); Methodology (equal); Writing‐review & editing (equal). **Jian Wang:** Conceptualization (lead); Funding acquisition (lead); Project administration (equal); Supervision (equal); Writing‐review & editing (equal). **Jingjing Wu:** Conceptualization (lead); Funding acquisition (lead); Project administration (lead); Supervision (lead); Writing‐original draft (lead); Writing‐review & editing (equal).

## Supporting information

Figure S1

Figure S2

Table S1‐S4

## Data Availability

The data sets used and/or analysed during the current study are available from the corresponding author upon reasonable request.
