# Supplementary figures and images for: PRMT5 promotes colorectal cancer growth by interaction with MCM7
Source: J Cell Mol Med. 2021 Mar 6;25(7):3537–47. doi: 10.1111/jcmm.16436 (PMC8034445; doi:10.1111/jcmm.16436)

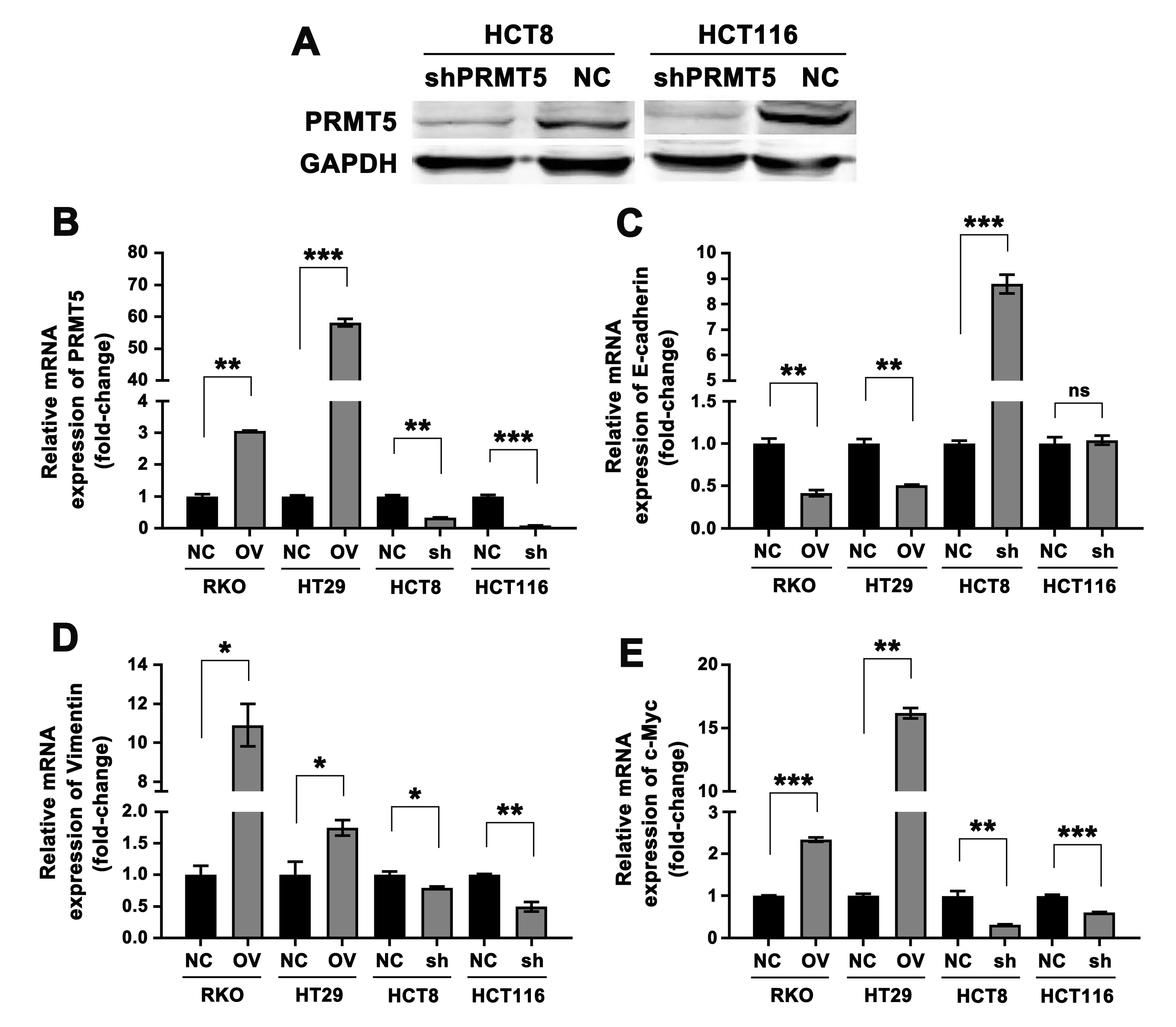

Supplement: Supplementary file 1 — Figure S1 [file JCMM-25-3537-s003.jpg]

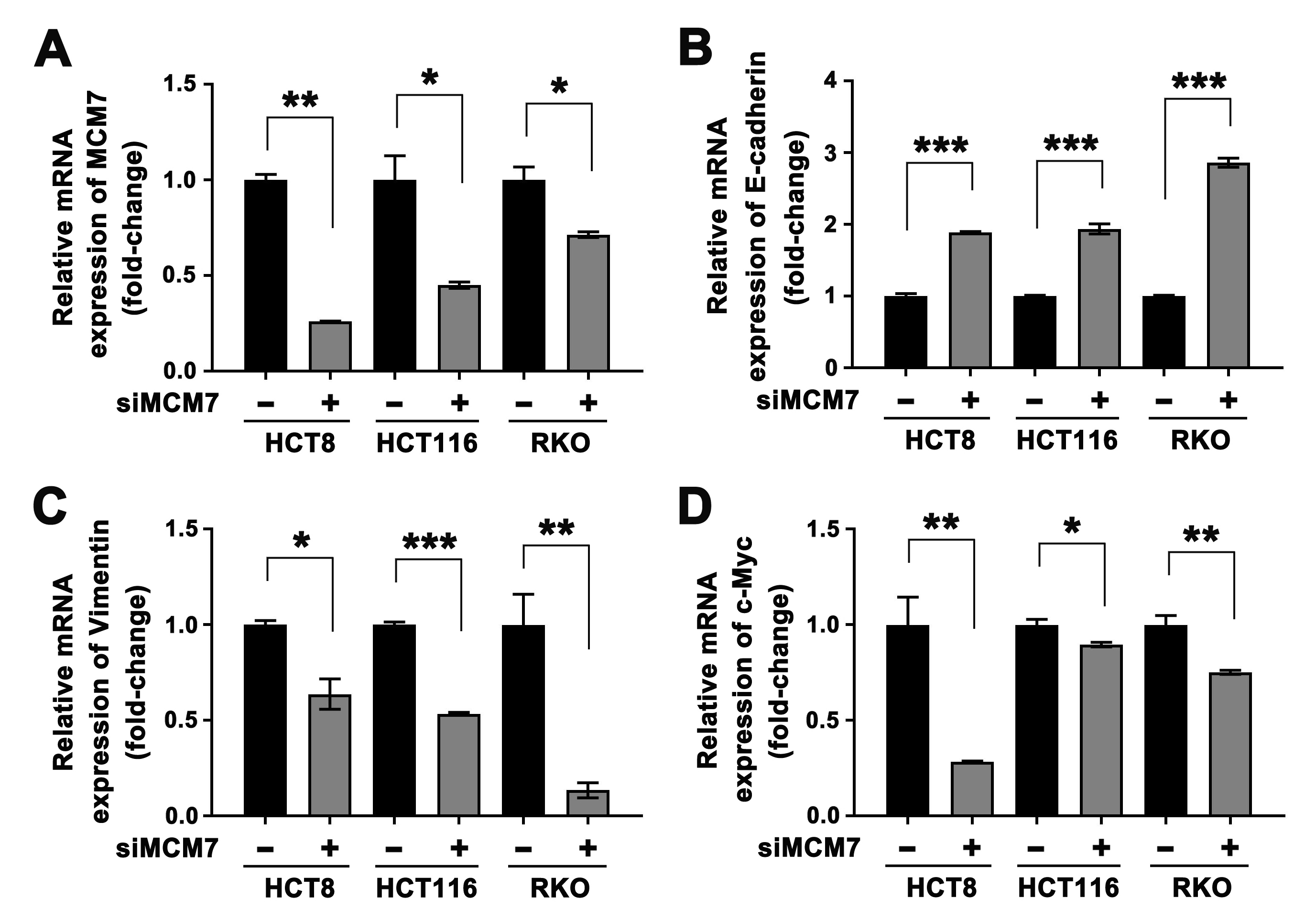

Supplement: Supplementary file 2 — Figure S2 [file JCMM-25-3537-s001.jpg]
