# Supplementary material for: PRMT5 promotes colorectal cancer growth by interaction with MCM7
Source: J Cell Mol Med. 2021 Mar 6;25(7):3537–47. doi: 10.1111/jcmm.16436 (PMC8034445; doi:10.1111/jcmm.16436)
Supplement: Supplementary file 3 — Table S1‐S4 [file JCMM-25-3537-s002.docx]

**Supplementary Materials and Methods**

**Western blotting analysis**

After cell lysates were prepared in ice-cold NP40 buffer with protease inhibitors for 30 min, protein extracts (30 µg per lane) were loaded onto 10-15% SDS-PAGE gels. Then, they were transferred to PVDF membranes (Bio-Rad, Hercules, CA, USA), which were subsequently incubated with the indicated primary antibodies against PRMT5 (rabbit, ab109451, 1:5000, Abcam, Cambridge, MA, USA), MCM7 (rabbit, ab52489, 1:5000, Abcam, Cambridge, MA, USA), FLAG (mouse, F1804, 1:2500, Sigma, St. Louis, MO, USA), Myc (rabbit, ab9106, 1:2500, Abcam, Cambridge, MA, USA) and GAPDH (rabbit, ab181602, 1:5000, Abcam, Cambridge, MA, USA). Proteins were visualized with the Odyssey Infrared Imaging System (Li-COR, Lincoln, NE, USA).

**IHC analysis**

Paraffin-embedded sections were deparaffinized with xylene, hydrated in ethanol and blocked with 0.3% hydrogen peroxide. The slides were then incubated with primary antibodies against PRMT5 (rabbit, ab109451, 1:200, Abcam, Cambridge, MA, USA) and MCM7 (rabbit, ab52489, 1:500, Abcam, Cambridge, MA, USA). Image-Pro Plus 6.0 software (Media Cybernetics, Rockville, MD, USA) was used to measure the relative protein expressions. All images were photographed at the same conditions using the same microscope (Leica, Cambridge, UK). Three fields were randomly selected on each section. The protein expression quantitation was analyzed by the integral optical density (IOD) and the area. We then calculated the qualifying scores using IOD/area. The average score of the three fields represents the mean densitometry of protein expression. Here, the score of PRMT5 expression < 95 was considered as low expression, whereas score ≥ 95 as high expression.

**Cell proliferation assay**

Cells were seeded in 96-well plates at 37°C in a humidified 5% CO_2_ incubator overnight. Subsequently, the proliferation of cells was determined by Cell Counting Kit-8 (CCK-8, Dojindo, Kumamoto, Japan) assay according to the manufacturer′s instruction and then detected at 450 nm using a spectrophotometer at the indicated time points. All experiments were repeated in triplicate for each sample.

**Cell migration and invasion assay**

The migration of cells was assessed using 24-well transwell chambers (8-μm pores, BD Biosciences, San Jose, CA, USA), whereas the invasion of cells was determined by 24-well invasion chambers coated with Matrigel (BD Biosciences, San Jose, CA, USA). Cells were seeded onto the upper chambers for 24 h in a humidified 5% CO_2_ incubator at 37°C. The migrated and invaded cells on the bottom surface of the chambers were then fixed in formalin, and stained with crystal violet (Sigma, St. Louis, MO, USA). Three random fields were counted under a light microscope. All experiments were done in triplicates.

**Cell cycle analysis**

Cells were seeded in triplicates in 6-well plates at 37°C in a humidified 5% CO_2_ incubator overnight, and then transiently transfected with siRNAs or controls as indicated. 48 hours later, cells were harvested by trypsin and fixed with ice-cold 70% ethanol for 24 hours. Next day, cells were stained with propidium iodide (PI, BD Biosciences, San Jose, CA, USA) and then subjected to flow cytometric analysis on a flow cytometer (Beckman Coulter, Brea, CA, USA). All experiments were carried out at least three times.

**Bioinformatics analysis**

The mRNA expression data in CRC and adjacent normal tissues for *PRMT5* and *MCM7* were extracted from The Cancer Genome Atlas (TCGA) data portal (freely accessible at <https://cancergenome.nih.gov/>). In addition, the NCBI Gene Expression Omnibus (GEO) databases (freely available at <https://www.ncbi.nlm.nih.gov/gds/>) were also used to analyze *PRMT5* and *MCM7* mRNA expression in CRC samples and normal controls with the accession numbers GSE21510, GSE24514, GSE22598, GSE31737, GSE89076, GSE20842, GSE44861, GSE33113, GSE60331, GSE89287, GSE23878 and GSE50421.

**Supplementary Table 1 Primers used for plasmid construction**

| Primer |  | | Sequences 5’—3’ | |
| --- | --- | --- | --- | --- |
| PRMT5 for coding sequence | | F | | 5’-tgctggaattctgcagatatcATGGCGGCGATGGCGGTC-3’ |
|  | | R | | 5’-ccctctagatgcatgctcgagCTAGAGGCCAATGGTATATGAGCG-3’ |
| Myc-PRMT5 FL | | F | | 5’-accgagctcggatccgaattcATGGCGGCGATGGCGGTC-3’ |
|  | | R | | 5’-ctcgggccctctagactcgagCTAGAGGCCAATGGTATATGAGCG-3’ |
| FLAG-MCM7 FL | | F | | 5’-tgctggaattctgcagatatcATGGCACTGAAGGACTACGCG-3’ |
|  | | R | | 5’-ccctctagatgcatgctcgagTCAGACAAAAGTGATCCGTGTCC-3’ |
| FLAG-MCM7 (1-248) | | F | | 5’-tgctggaattctgcagatatcATGGCACTGAAGGACTACGCG-3’ |
|  | | R | | 5’-ccctctagatgcatgctcgagTCAATTTCCCACAGGCACCT-3’ |
| FLAG-MCM7 (249-427) | | F | | 5’-tgctggaattctgcagatatcATCCCTCGTAGTATCACGGTGC-3’ |
|  | | R | | 5’-ccctctagatgcatgctcgagTCACAGTTCTCCACTCACGGAGT-3’ |
| FLAG-MCM7 (428-719) | | F | | 5’-tgctggaattctgcagatatcACCTTAGAGGGTGGGGCCC-3’ |
|  | | R | | 5’-ccctctagatgcatgctcgagTCAGACAAAAGTGATCCGTGTCC-3’ |

**Supplementary Table 2 Primers used for qRT-PCR**

| Primer |  | | Sequences 5’—3’ | |
| --- | --- | --- | --- | --- |
| PRMT5 | | F | | 5’-TTGCCGGCTACTTTGAGACT-3’ |
|  | | R | | 5’-ACAGATGGTTTGGCCTTCAC-3’ |
| MCM7 | | F | | 5’-CCTACCAGCCGATCCAGTCT-3’ |
|  | | R | | 5’-CCTCCTGAGCGGTTGGTTT-3’ |
| E-cadherin | | F | | 5’-AGGCCAAGCAGCAGTACATT-3’ |
|  | | R | | 5’-CATTCACATCCAGCACATCC-3’ |
| Vimentin | | F | | 5’-CGAAACTTCTCAGCATCACG-3’ |
|  | | R | | 5’-GCAGAAAGGCACTTGAAAGC-3’ |
| c-Myc | | F | | 5’-TTCGGGTAGTGGAAAACCAG-3’ |
|  | | R | | 5’-CAGCAGCTCGAATTTCTTCC-3’ |
| GAPDH | | F | | 5’-CCCTTCATTGACCTCAACTACATG-3’ |
|  | | R | | 5’-TGGGATTTCCATTGATGACAAGC-3’ |

**Supplementary Table 3 List of PRMT5-interacting proteins identified by Co-IP and MS analysis**

| Protein | Description | Mw (kDa) | Score |
| --- | --- | --- | --- |
| KIF11 | kinesin family member 11 | 119.0850394 | 124.9788826 |
| WDR77 | WD repeat domain 77 | 36.70107605 | 79.73748469 |
| MYO1C | myosin IC | 118.9203949 | 42.83775854 |
| HNRNPH1 | heterogeneous nuclear ribonucleoprotein H1 | 49.19841117 | 41.18944931 |
| HSPA8 | heat shock protein family A (Hsp70) member 8 | 68.76318831 | 32.39485168 |
| PRPF6 | pre-mRNA processing factor 6 | 106.8578002 | 30.01375246 |
| MCM3 | minichromosome maintenance complex component 3 | 90.9240309 | 27.59415531 |
| ARHGAP23 | Rho GTPase activating protein 23 | 125.6317717 | 26.75810373 |
| PKP3 | plakophilin 3 | 87.02899667 | 26.38475239 |
| GANAB | glucosidase II alpha subunit | 106.8066523 | 26.33135509 |
| SPTAN1 | spectrin alpha | 284.3642231 | 25.63142598 |
| MCM7 | minichromosome maintenance complex component 7 | 81.25657355 | 25.08548868 |
| RPL4 | ribosomal protein L4 | 47.66743012 | 24.32178473 |
| PPM1B | protein phosphatase, Mg2+/Mn2+ dependent 1B | 41.80331706 | 22.40043604 |
| ACTC1 | actin, alpha, cardiac muscle 1 | 41.99188264 | 21.43938828 |
| HNRNPH2 | heterogeneous nuclear ribonucleoprotein H2 | 49.23228927 | 21.22136235 |
| TRIM28 | tripartite motif containing 28 | 79.4234891 | 19.74555731 |
| RPL7 | ribosomal protein L7 | 24.41738789 | 19.51952219 |
| RPS8 | ribosomal protein S8 | 24.19016371 | 19.18566906 |
| MCM5 | minichromosome maintenance complex component 5 | 77.54373204 | 19.07340395 |
| EIF4A3 | eukaryotic translation initiation factor 4A3 | 46.84118434 | 18.78489459 |
| PFKP | phosphofructokinase, platelet | 85.26145654 | 18.65190649 |
| TUBA1A | tubulin alpha 1a | 46.26784264 | 17.90246367 |
| ENO1 | enolase 1 | 47.13932161 | 16.75494599 |
| SPTBN1 | Spectrin beta | 274.4386972 | 16.21052551 |
| HNRNPF | heterogeneous nuclear ribonucleoprotein F | 45.64285734 | 14.88946676 |
| SBSN | suprabasin | 60.50459897 | 14.27737331 |
| PCBP1 | poly(rC) binding protein 1 | 37.47394517 | 13.78527164 |
| HSP90B1 | heat shock protein 90 beta family member 1 | 92.41134842 | 12.97144508 |
| MCM6 | minichromosome maintenance complex component 6 | 92.83124152 | 12.42669046 |
| RIOK1 | RIO kinase 1 | 65.54166349 | 12.0832684 |
| CAPRIN1 | cell cycle associated protein 1 | 76.81431688 | 11.98828375 |
| RBM10 | RNA binding motif protein 10 | 94.31221265 | 11.73186779 |
| LIMA1 | LIM domain and actin binding 1 | 85.1733623 | 11.68043828 |
| GEMIN4 | gem nuclear organelle associated protein 4 | 118.7454004 | 11.42883694 |
| NPEPPS | aminopeptidase puromycin sensitive | 102.9221832 | 11.03093958 |
| RNH1 | ribonuclease/angiogenin inhibitor 1 | 49.94108966 | 10.89783597 |
| CAPN1 | calpain 1 | 81.8381919 | 10.67668223 |
| RPL7A | ribosomal protein L7a | 21.53140826 | 10.56866407 |
| MTHFD1 | methylenetetrahydrofolate dehydrogenase, cyclohydrolase and formyltetrahydrofolate synthetase 1 | 101.4953107 | 10.04227567 |
| HSPA7 | heat shock protein family A (Hsp70) member 7 | 40.21958921 | 9.948091507 |
| PPL | Periplakin | 131.2957541 | 9.911823988 |
| OAS3 | 2'-5'-oligoadenylate synthetase 3 | 121.0930254 | 9.274558067 |
| EIF4G1 | eukaryotic translation initiation factor 4 gamma 1 | 154.7089547 | 9.212411404 |
| EIF3F | eukaryotic translation initiation factor 3 subunit F | 37.54015324 | 9.237268448 |
| HNRNPC | heterogeneous nuclear ribonucleoprotein C (C1/C2) | 27.8044595 | 8.560582399 |
| EPS8L2 | EPS8 like 2 | 80.57012574 | 8.325411677 |
| DDX20 | DEAD-box helicase 20 | 92.1825092 | 8.043458939 |
| KHSRP | KH-type splicing regulatory protein | 72.98228065 | 7.943405628 |
| SNRNP200 | small nuclear ribonucleoprotein U5 subunit 200 | 244.3526288 | 7.89485836 |
| MOGS | mannosyl-oligosaccharide glucosidase | 80.65262951 | 7.798856258 |
| ABCE1 | ATP binding cassette subfamily E member 1 | 67.2710207 | 7.843206644 |
| AZGP1 | alpha-2-glycoprotein 1, zinc-binding | 34.23710609 | 7.643528461 |
| TFRC | transferrin receptor | 84.81794733 | 7.605732441 |
| EPPK1 | Epiplakin | 552.7906845 | 7.585525274 |
| CTNND1 | catenin delta 1 | 90.22870024 | 7.571652174 |
| EEF2 | eukaryotic translation elongation factor 2 | 95.27695382 | 7.564430475 |
| TPM4 | tropomyosin 4 | 28.5044925 | 7.476547718 |
| RPL8 | ribosomal protein L8 | 28.00729016 | 7.443237066 |
| UQCRC1 | ubiquinol-cytochrome c reductase core protein I | 52.61243235 | 7.286114931 |
| CNP | 2',3'-cyclic nucleotide 3' phosphodiesterase | 45.07033459 | 7.230605245 |
| EHD4 | EH domain containing 4 | 61.1365258 | 6.990188956 |
| PSMC5 | proteasome 26S subunit, ATPase 5 | 44.75569136 | 6.920660973 |
| SERPINB3 | serpin family B member 3 | 44.53654692 | 6.815636158 |
| YWHAH | tyrosine 3-monooxygenase/tryptophan 5-monooxygenase activation protein eta | 28.20101996 | 6.611038089 |
| AZGP1 | alpha-2-glycoprotein 1, zinc-binding | 26.33719102 | 6.367825866 |
| HNRNPA2B1 | heterogeneous nuclear ribonucleoprotein A2/B1 | 35.98387254 | 6.260573387 |
| DHX15 | DEAH-box helicase 15 | 90.87516973 | 5.91312933 |
| SRPRB | SRP receptor beta subunit | 29.68376282 | 5.881013036 |
| RPL13 | ribosomal protein L13 | 24.24652727 | 5.837415218 |
| AP2M1 | adaptor related protein complex 2 mu 1 subunit | 49.35791022 | 5.790001631 |
| PKP1 | plakophilin 1 | 80.44545865 | 5.599975228 |
| MRPS34 | mitochondrial ribosomal protein S34 | 25.63448963 | 5.432547808 |
| ASS1 | argininosuccinate synthase 1 | 46.5009656 | 2.067810535 |
| SERPINB4 | serpin family B member 4 | 42.47131131 | 1.945904493 |

**Supplementary Table 4 Details of GEO datasets used in this study**

| GEO | Country | Adjacent normal tissue (N) | CRC (N) | First author (publication year) |
| --- | --- | --- | --- | --- |
| GSE21510 | Japan | 25 | 123 | Tsukamoto S (2011) |
| GSE24514 | Finland | 15 | 34 | Alhopuro P (2012) |
| GSE22598 | Japan | 17 | 17 | Okazaki S (2012) |
| GSE31737 | USA | 40 | 40 | Loo LW (2012) |
| GSE89076 | Japan | 39 | 41 | Satoh K (2017) |
| GSE20842 | Germany | 65 | 65 | Gaedcke J (2010) |
| GSE44861 | USA | 55 | 56 | Ryan BM (2014) |
| GSE33113 | Netherlands | 6 | 90 | Kemper K (2012) |
| GSE60331 | Belgium | 19 | 31 | Verstraete M (2015) |
| GSE89287 | Netherlands | 17 | 54 | Zuurbier L (2017) |
| GSE23878 | Saudi Arabia | 24 | 35 | Uddin S (2011) |
| GSE50421 | Saudi Arabia | 25 | 24 | AI Mahri S (2020) |

**Supplementary Figure Legends**

Figure S1 A, Western blot analysis of PRMT5 levels in HCT8 and HCT116 cells stably transfected with the control or PRMT5-specific shRNA (HCT8-NC/HCT8-shPRMT5 and HCT116-NC/HCT116-shPRMT5). B-E, qRT-PCR analysis of (B) PRMT5, (C) E-cadherin, (D) Vimentin and (E) c-Myc mRNA expressions in the RKO-NC/RKO-PRMT5, HT29-NC/HT29-PRMT5, HCT8-NC/HCT8-shPRMT5 and HCT116-NC/HCT116-shPRMT5 cells. Gene mRNA expression was normalized to GAPDH. Data were presented as the mean ± SEM of experiments performed in triplicate. **P* < 0.05; ***P* < 0.01; ****P* < 0.001; ns, no significance.

Figure S2 qRT-PCR analysis of (A) MCM7, (B) E-cadherin, (C) Vimentin and (D) c-Myc mRNA expressions in the HCT8, HCT116 and RKO cells transfected with the scramble control or siMCM7. Gene mRNA expression was normalized to GAPDH. Data were presented as the mean ± SEM of experiments performed in triplicate. **P* < 0.05; ***P* < 0.01; ****P* < 0.001.
